# Autoimmune Hepatitis with Anti Centromere Antibodies

**DOI:** 10.1155/2013/742080

**Published:** 2013-07-24

**Authors:** Moushumi Lodh, Debkant Pradhan, Ashok Parida

**Affiliations:** ^1^Department of Biochemistry, The Mission Hospital, Durgapur, West Bengal 713212, India; ^2^Department of Microbiology, The Mission Hospital, Durgapur, West Bengal 713212, India; ^3^Department of Cardiology, The Mission Hospital, Durgapur, West Bengal 713212, India

## Abstract

We present the case report of a 49-year-old type 2 diabetes mellitus patient presenting with abdominal pain and black stool for 15 days. A proper workup of laboratory investigations helped us diagnose autoimmune hepatitis with anticentromere antibodies. The authors would like to highlight that screening AIH patients for anticentromere antibody is not mandatory but can be considered, especially in the presence of disease-related symptomatology for quicker, more accurate diagnosis and optimum management.

## 1. Introduction

Autoimmune liver disease is not an uncommon cause of chronic hepatitis in women. Although autoimmune destruction usually occurs without an identifiable trigger, it is generally a progressive hepatitis with increased immunoglobulins and autoantibodies, which primarily responds to immunosuppression.

## 2. Case Report

**Figure 1:**
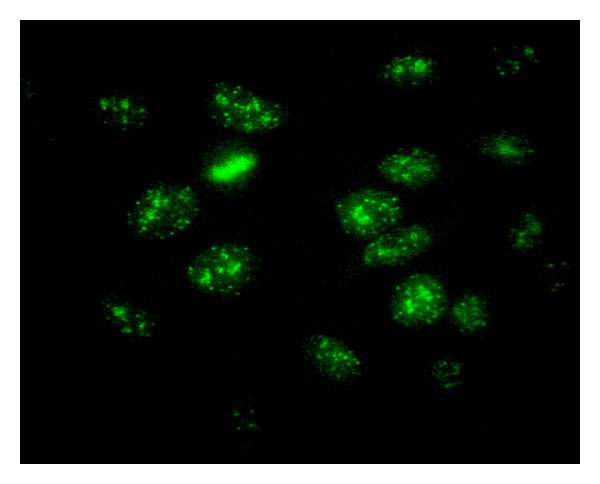
Indirect immunofluorescence on HEp-2 cells performed with an autoimmune hepatitis serum and demonstrating centromere staining.

A 49-year-old lady presented with history of mild, intermittent abdominal pain of 15 days duration associated with passage of black colored stool, nausea, loss of appetite, and generalized weakness. At admission, she was pale, afebrile, with pulse 110/min, blood pressure 150/90 mm Hg, respiratory rate 26/min, and random plasma glucose 230 mg/dL. There was dyspnea on exertion. Skin was warm with no rash or discoloration. Her abdomen was soft, and bowel sounds were audible. There was a generalized abdominal tenderness with an irregular lump near the epigastrium. The patient was conscious and well oriented with no neurological deficit. She has undergone percutaneous transluminal coronary angioplasty (PTCA) to the right coronary artery 8 years back. The patient had no history of alcohol abuse or received drugs that can idiosyncratically cause hepatitis. Laboratory investigations were as follows (reference ranges in parentheses): hemoglobin 9.1 g% (12–15), PCV 28.2% (36–46), total count 7000/cumm (4000–10,000), RBC 3.27 million/cumm (4.5–5.5), platelet 1.59 lakhs/cumm (1.5–4), total bilirubin 1.8 mg/dL (upto 1), direct bilirubin 0.8 mg/dL (upto 0.3), glycosylated hemoglobin 10.7% (6–8), total protein 5.7 g/dL (6.5–8.1), albumin 2.4 g/dL (3.5–5), alanine transaminase 257 U/L (0–31), aspartate transaminase 224 U/L (0–32), alkaline phosphatase 793 U/L (30–279), gamma glutamyl transferase 477 U/L (1–94), lipase 96 U/L (upto 160), amylase 48 U/L (25–125), lactic dehydrogenase 1203 U/L (266–500), and prothrombin time 18 seconds (control 11.5) INR 1.58. Urea, creatinine, alpha-1 antitrypsin, serum copper, and electrolytes were within reference range. Viral serologies for antibodies to hepatitis B surface antigen, antihepatitis B surface antigen, antihepatitis B core antigen, antihepatitis C virus, cytomegalovirus, Epstein-Barr virus, herpes simplex virus, and human immunodeficiency virus were all negative. Immunoglobulin G was 1987 mg/dL (700–1600 mg/dL). Antinuclear antibody (ANA) by IFA (1 : 320 titer) on Hep-2 cells (HEp-2000 IgG fluorescent ANA-Ro test system, Immunoconcepts, USA) revealed anticentromere antibodies (Figure 1) showing 40–60 discrete speckles distributed over the nucleus, either dispersed or gathered closely together on the chromosomes of cells undergoing division. Four positive ANA controls (homogeneous, speckled, centromere, and nucleolar) included in the kit were also run for comparison. ANA repeated by enzyme immunoassay was 195.6 units (<20). Immunochromatography showed centromere B and soluble liver antigen/liver-pancreas antigen (SLA/LP) antibodies to be positive. Antithyroid antibodies (antiperoxidase and antithyroglobulin) and antigastric parietal cell antibodies were not detected by line immunoassay. Liver biopsy showed a portal mononuclear cell infiltration, interface hepatitis in the liver tissue, and bridging fibrosis. International autoimmune hepatitis group score was 16. Upper gastrointestinal endoscopy revealed erosive pangastritis with duodenal erosions (D1 and D2). Rapid urease test for *Helicobacter pylori* was negative. Ultrasonography of the whole abdomen was a normal study. Echocardiography revealed severe mitral regurgitation and mild pericardial effusion. Based on all these findings, diagnosis of autoimmune hepatitis with type 2 diabetes mellitus, coagulopathy, and ischemic heart disease was made. The absence of piecemeal necrosis or florid bile duct lesion along with antismooth muscle antibody (ASMA) and antimitochondrial antibody (AMA) negativity ruled out autoimmune hepatitis-primary biliary cirrhosis (AIH/PBC) overlap syndrome. Injection insulin H Mixtard (50 : 50) 16 units thirty minutes before breakfast, 22 units thirty minutes before lunch, and 14 units before dinner were started. She was put on diabetic diet (1500 kcal/day). Prednisolone 30 mg daily was started in combination with azathioprine 50 mg daily. She was discharged after 7 days in a stable condition with medical advice (pantocid 40 mg once a day (O. D) for 4 weeks, ecosprin 150 mg O. D, cardace 10 mg O. D) and to continue insulin and steroids. At follow up after 4 weeks, her liver enzymes had reduced to within reference range, but ANA still tested positive at 1 : 160 titer. Random plasma glucose was 140 mg/dL; she did not develop any complication due to steroid therapy.

## 3. Discussion

Autoimmune hepatitis (AIH) can present as an acute or even an alarmingly fulminant hepatitis or conversely be asymptomatic and recognized only incidentally by routine biochemical tests of liver function. The critical and readily measurable indices reflecting the essence of AIH are a lack of evidence of current viral infection, transferases greater than twice upper normal limit, and immunoglobulin G greater than 1.1 times upper normal limit, interface hepatitis with plasma cell prominence, and positivity to an acceptable titer for SMA, ANA, anti-SLA/LP, or anti-LKM [1]. Our case is noteworthy due to the apparently innocuous presentation and the presence of anticentromere antibody (ACA), which is commonly associated with the limited form of systemic sclerosis, primary biliary cirrhosis, and sometimes with diffuse form of systemic sclerosis. 

The immune response that targets the liver in AIH involves cytotoxic T lymphocytes, which damage the hepatocytes via the production of interleukins (IL-2, IL- 12, and tumor necrosis factor-*α* (TNF-*α*)). However, the molecular target of the T lymphocyte response has not yet been identified [2]. The Committee for Autoimmune Serology of the International Autoimmune Hepatitis Group (IAIHG) provided guidelines on testing for autoantibodies relevant to AIH and concluded that indirect immunofluorescence assay (IFA) on fresh sections of multiorgan (liver, kidney, and stomach) from rodents (usually rat) should be the first line screening and the use of the three tissues enabling simultaneous detection of virtually all the autoantibodies relevant to liver disease, namely, against smooth muscle antigen (SMA), Antinuclear antibody (ANA), anti-liver-kidney microsome (LKM1), antimitochondrial antibody (AMA), and anticytosolic liver antigen type 1 (LC1) [3]. Despite its limited clinical sensitivity of 7–19%, the testing for anti-SLA antibodies can be considered as pathognomonic markers of AIH, with specificity close to 100% [4]. AIH may be due to dysfunction of cellular and humoral immunity related to systemic sclerosis as anticentromere antibody has been detected in 13% of patients with AIH [5]. Twenty percent of patients of AIH can have other autoimmune diseases such as Hashimotos thyroiditis, type 1 diabetes, rheumatoid arthritis, systemic lupus erythematosus, ulcerative colitis/Crohn's disease, and celiac disease [6]. Our patient had type 2 diabetes mellitus. No significant differences in age, sex, and onset pattern of the disease, progression to hepatic failure, and relapse rate were present between ACA-AIH and other AIH groups [7]. 

Autoimmune hepatitis is one of the few liver diseases with excellent response to therapy; most patients with AIH have a favorable response to treatment with prednisolone and azathioprine, although some patients with refractory AIH or more aggressive disease require more potent immune-suppressant agents, such as cyclosporine [2]. Patients without cirrhosis who undergo treatment have a 10–20 year survival probability more than 80% [6]. Screening AIH patients for anticentromere antibody is not mandatory but can be considered, especially in the presence of disease-related symptomatology for quicker, more accurate diagnosis and optimum management.
